# Localization and Transcriptional Responses of *Chrysoporthe austroafricana* in *Eucalyptus grandis* Identify Putative Pathogenicity Factors

**DOI:** 10.3389/fmicb.2016.01953

**Published:** 2016-12-08

**Authors:** Ronishree Mangwanda, Lizahn Zwart, Nicolaas A. van der Merwe, Lucy Novungayo Moleleki, Dave Kenneth Berger, Alexander A. Myburg, Sanushka Naidoo

**Affiliations:** ^1^Department of Genetics, Forestry and Agricultural Biotechnology Institute, Genomics Research Institute, University of PretoriaPretoria, South Africa; ^2^Department of Microbiology and Plant Pathology, Forestry and Agricultural Biotechnology Institute, University of PretoriaPretoria, South Africa; ^3^Department of Plant and Soil Sciences, Forestry and Agricultural Biotechnology Institute, Genomics Research Institute, University of PretoriaPretoria, South Africa

**Keywords:** fungal pathogenicity, dual RNA-sequencing, cell wall degrading enzymes, hormone signaling

## Abstract

*Chrysoporthe austroafricana* is a fungal pathogen that causes the development of stem cankers on susceptible *Eucalyptus grandis* trees. Clones of *E. grandis* that are partially resistant and highly susceptible have been identified based on the extent of lesion formation on the stem upon inoculation with *C. austroafricana.* These interactions have been used as a model pathosystem to enhance our understanding of interactions between pathogenic fungi and woody hosts, which may be different to herbaceous hosts. In previous research, transcriptomics of host responses in these two clones to *C. austroafricana* suggested roles for salicylic acid and gibberellic acid phytohormone signaling in defense. However, it is unclear how the pathogen infiltrates host tissue and which pathogenicity factors facilitate its spread in the two host genotypes. The aim of this study was to investigate these two aspects of the *E. grandis–C. austroafricana* interaction and to test the hypothesis that the pathogen possesses mechanisms to modulate the tree phytohormone-mediated defenses. Light microscopy showed that the pathogen occurred in most cell types and structures within infected *E. grandis* stem tissue. Notably, the fungus appeared to spread through the stem by penetrating cell wall pits. In order to understand the molecular interaction between these organisms and predict putative pathogenicity mechanisms of *C. austroafricana*, fungal gene expression was studied *in vitro* and *in planta*. Fungal genes associated with cell wall degradation, carbohydrate metabolism and phytohormone manipulation were expressed *in planta* by *C. austroafricana*. These genes could be involved in fungal spread by facilitating cell wall pit degradation and manipulating phytohormone mediated defense in each host environment, respectively. Specifically, the *in planta* expression of an *ent-kaurene oxidase* and *salicylate hydroxylase* in *C. austroafricana* suggests putative mechanisms by which the pathogen can modulate the phytohormone-mediated defenses of the host. These mechanisms have been reported in herbaceous plant–pathogen interactions, supporting the notion that these aspects of the interaction are similar in a woody species. This study highlights *ent-kaurene oxidase* and *salicylate hydroxylase* as candidates for further functional characterization.

## Introduction

The value of model plant–pathogen systems for studying the complexities of plant defense is undeniable. However, most of the current knowledge regarding plant defense originates from research conducted in established model systems with herbaceous plants such as *Nicotiana* spp. and *Arabidopsis thaliana* (Tsuda and Somssich, 2015), and model systems with woody perennials are scarce. To expand this niche, the interaction of *Eucalyptus grandis* and *Chrysoporthe austroafricana* has been established as a model system.

The fungal pathogen *C. austroafricana* causes the development of stem cankers on susceptible *E. grandis* trees (Roux et al., 2003). It was first discovered in the late 1980s (Wingfield et al., 1989). The pathogen was previously described as *Chrysoporthe cubensis*, but reclassified when phylogenetic and morphological analyses showed that it was a different species (Myburg et al., 2002; Gryzenhout et al., 2004). In South Africa, *C. austroafricana* may have undergone a host shift from native *Syzygium* spp. to non-native *Eucalyptus* (Heath et al., 2006; Wingfield et al., 2008). Damage to plantations caused by this pathogen has largely been limited through the use of *E. grandis x E. urophylla* hybrids that exhibit increased disease resistance.

*Chrysoporthe austroafricana* is pathogenic on *E. grandis* and exhibits different levels of virulence in the clones TAG5 and ZG14, with longer lesions in ZG14 compared to TAG5 (van Heerden et al., 2005; Naidoo et al., 2013). The clone ZG14 has high mortality rates after infection, while lesions are confined in TAG5. Therefore, the clones are considered highly susceptible and moderately resistant to the pathogen, respectively. This pathosystem has been exploited as a model system to study host–pathogen interactions in woody plants. Very little is known about the physical location and pathogenicity mechanisms of *C. austroafricana* within this host. The spread of wood-rotting fungi and various host defense mechanisms against them have been described (Pearce, 1996). Some of these fungi are able to spread by degrading cell walls, while those that lack this ability spread through weak points in the cell wall like pits and perforation plates (Pearce, 1996). However, to our knowledge, the spread of non-wood-rotting putative necrotrophs that proliferate in woody stem tissue has not been described. Pathogen spread would also be influenced by the response of the host to the pathogenicity strategy of the pathogen. Given the development of longer lesions on the stems of ZG14 compared to TAG5 (Naidoo et al., 2013), we hypothesize that pathogen spread would be faster in ZG14 than in TAG5.

In previous work, we examined the expression and hormone profiles of TAG5 and ZG14 in response to challenge with *C. austroafricana* at 3 days post-inoculation (Naidoo et al., 2013; Mangwanda et al., 2015). While genes involved in cell wall modifications and response to oxidative stress were common between the hosts, hormone signaling seemed to facilitate resistance in TAG5. One of the main findings was that basal levels of salicylic acid were significantly higher in TAG5 compared to ZG14, with SA marker gene expression profiles supporting this finding. TAG5 displayed a decrease in gibberellic acid (GA) levels following inoculation and this phenomenon was not apparent in ZG14 (Mangwanda et al., 2015). This initial investigation revealed that the woody perennial had a similar defense mechanism to that found in herbaceous systems. For example, it was found that high levels of SA contribute to resistance in tobacco (Verberne et al., 2000) and that GA has a negative effect on resistance in rice (Qin et al., 2012; Yang et al., 2016). Furthermore, the results suggest that *C. austroafricana* encounters two different host environments, and we hypothesize that the fungus employs different mechanisms to respond to the phytohormone signaling and other defenses in each host.

Previous studies showed that the stress induced by growing the pathogen *in vitro* on nutrient limiting media can result in the expression of genes associated with pathogenicity (Hottes et al., 2004; Bruno et al., 2010). The rationale is that the nutrient limiting medium presents a stressful environment for the pathogen, a proxy for the harsh *in planta* environment that it would encounter during the early stages of infection, and would thus induce similar pathogenicity mechanisms. Apart from *in vitro* assays, *in planta* expression of pathogenicity genes can be studied using dual RNA-sequencing, a powerful tool for capturing responses of the pathogen in direct contact with host tissue (Westermann et al., 2012; Hayden et al., 2014).

The aim of this study was to examine putative mechanisms by which *C. austroafricana* causes disease in *E. grandis.* To achieve this, we tracked pathogen spread using light microscopy and studied the transcriptional responses of the pathogen within the two host environments.

## Materials and Methods

### Microscopy

Stems of 2–3 year old *E. grandis* clones TAG5 and ZG14, which are moderately resistant and highly susceptible, respectively, were inoculated with *C. austroafricana* as described previously (Mangwanda et al., 2015). A 5 mm diameter section of bark was removed with a cork borer 30 cm above the base of the stem. The plant was inoculated by inserting an agar plug covered in actively growing mycelium of the type isolate of *C. austroafricana* (Forestry and Agricultural Biotechnology Institute culture collection, CMW2113; Centraalbureau voor Schimmelcultures KNAW Fungal Biodiversity Center – CBS 112916, Agricultural Research Council National Collection of Fungi – PREM 58023, dried culture) in the wound. The section of bark was placed on the agar plug and the wound was sealed with Parafilm (Sigma-Aldrich). After 3, 7, 14, 21, and 42 days, small pieces of *E. grandis* stem were excised at the inoculation site, trimmed and fixed in FAA (45% ethanol, 5% acetic acid, and 5% formalin in distilled water). The samples were sectioned with a sliding microtome (approximately 15 μm thick) or dehydrated with a butanol series, embedded in paraffin wax (Sigma-Aldrich, Supplementary Table S7) and sectioned with a rotary microtome (8–10 μm thick). Sections were stained with Safranin O (uniLAB) and Fast Green FCF (uniLAB) and mounted in Entellan (Merck Millipore), or stained with and mounted in lactophenol blue solution (Fluka, Sigma-Aldrich).

### Transcriptome Sequencing of *C. austroafricana In vitro* and *In planta*

#### Culturing of *C. austroafricana In vitro*

**FIGURE 1:**
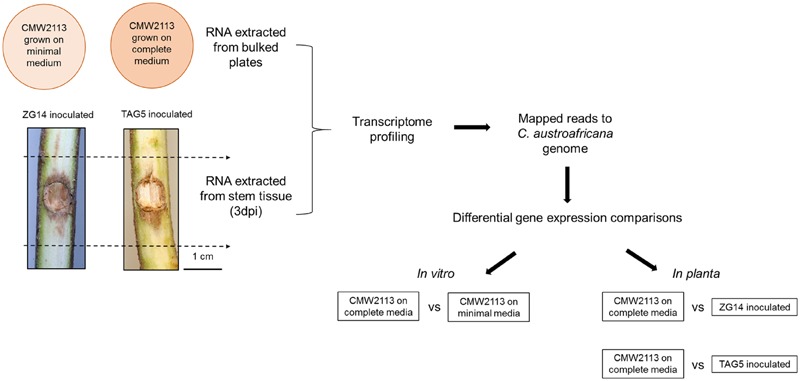
**Schematic representation of the transcriptome profiling performed for *Chrysoporthe austroafricana*.** CMW2113 represents the type isolate of *C. austroafricana*.

The type isolate of *C. austroafricana* (CMW2113) was cultured on complete and minimal media (Leslie, 2007) covered with a nylon membrane disk (Sigma-Aldrich, 0.45 μm). Plates (90 mm diameter) were incubated at 28°C until the mycelial mat covered the nylon membrane (**Figure 1**). Three independent biological replicates each were used for complete and minimal media. Mycelium was harvested, bulked and frozen in liquid nitrogen. RNA was extracted using the Plant/Fungi total RNA purification kit (Norgen Biotek, Sigma-Aldrich), treated with RNase-free DNaseI enzyme (Qiagen, Inc., Valencia, CA, USA) and purified using the RNeasy^®^ MinElute Kit (Qiagen, Inc., Valencia, CA, USA) according to the manufacturer’s protocol. RNA was sent to the Beijing Genome Institute (BGI) for TruSeq using the Illumina Genome Analyser with a 50 bp paired end module (Illumina, San Diego, CA, USA).

#### *In planta* Expression of *C. austroafricana* Genes

*Eucalyptus grandis* genotypes TAG5 and ZG14 were inoculated with *C. austroafricana* CMW2113 as described by Mangwanda et al. (2015). Inoculated and control stem material was harvested with three independent biological replicates consisting of three ramets each, at 3 days post-inoculation (**Figure 1**). RNA was extracted from these samples as indicated in Mangwanda et al. (2015) and sent to the BGI for transcriptome profiling using the Illumina Genome Analyser with a 50 bp paired end module (Illumina, San Diego, CA, USA).

### Bioinformatics Analysis

#### *C. austroafricana* Genome Annotation

Putative predictions for *C. austroafricana* gene models (GenBank JYIP00000000; Wingfield et al., 2015) were searched for with the BLASTX algorithm against the GenBank NR database (e-value > 1e-10) using Blast2GO (Conesa and Gotz, 2008). This software was additionally used to assign GO (Gene Ontologies) and KEGG (Kyoto Encyclopedia of Genes and Genomes) annotations to the predicted genes. Further putative functions such as pathogenicity, reduced virulence, lethality and increased virulence were assessed using a BLASTP (e-value > 1e-5) against the PHI-database (Winnenburg et al., 2006). A BLASTP analysis against the Carbohydrate-Active enZyme (CAZymes) database^1^ was performed with an e-value < 1e^-2^, a bit score threshold of 55 and a rule level of support of 40.

#### Read Mapping and Transcript Quantification

RNA reads obtained from BGI were analyzed using the Galaxy platform (Giardine et al., 2005; Blankenberg et al., 2010; Goecks et al., 2010). The quality of the reads was assessed using FASTQC v0.5 and FASTQ groomer v1.0.4. Mapping of the reads was performed against the *C. austroafricana* genome and *E. grandis* genome using the TopHat v2.0.4 package. Thereafter, Cuﬄinks v1.03 was used to quantify the transcript abundance.

#### Differential Gene Expression

Differential gene expression of the *in vitro* and *in planta* samples was determined using Cuffdiff v1.0.3 (FPKM > 100). The following comparisons were made: complete medium vs. minimal medium, complete medium vs. *C. austroafricana* genes expressed in TAG5 inoculated samples and complete medium vs. *C. austroafricana* genes expressed in ZG14 inoculated samples (**Figure 1**). GO enrichment analysis was performed using Fisher’s exact test (*p*-value < 0.05) in Blast2GO.

### Quantitative Real Time PCR Validation

Primers for quantitative polymerase chain reaction (qPCR) analysis were designed using Primer3 v0.4, and their specificity verified using NCBI-Primer BLAST to detect any cross-species hybridization. In addition, the primers were validated using a BLASTN algorithm in a local blast on CLC Genomics Workbench^2^ against the *C. austroafricana* genome and the genome of a well-known eucalypt endophyte, *Botryosphaeria dothidea*. First strand cDNA synthesis was performed using the Transcriptor First Strand cDNA Synthesis Kit (Roche, Mannheim, Germany) according to the manufacturer’s protocol.

Quantitative PCR was performed on the QuantStudio^TM^ 12K Flex Real-Time PCR System (Applied Biosystems^®^, Life Technologies, Inc.). The reaction consisted of the following reagents: primers (0.3 μM), SYBR^®^ Select Master Mix (2X, 5 μl), and cDNA (1 μl), in a total reaction of volume of 10 μl. All reactions were performed in triplicate. The following PCR protocol was used: 50°C for 2 min, 95°C for 10 min, 40 cycles of 95°C for 15 s and 58°C for 1 min. Melting curve analysis involved one cycle of 95°C for 15 s, 60°C for 1 min and a dissociation cycle of 95°C for 15 s. Relative expression and normalization were performed using *qBASE*plus v1.0. The candidate reference genes, g10189 (*unknown*) and g1986 (*vacuolar protein sorting-associated protein*) were stably expressed *in vitro* and *in planta*. The Student’s *t*-test (*p* < 0.05) was applied to test for significance between samples.

## Results

### Macroscopic Changes during Infection of *E. grandis* with *C. austroafricana*

**FIGURE 2:**
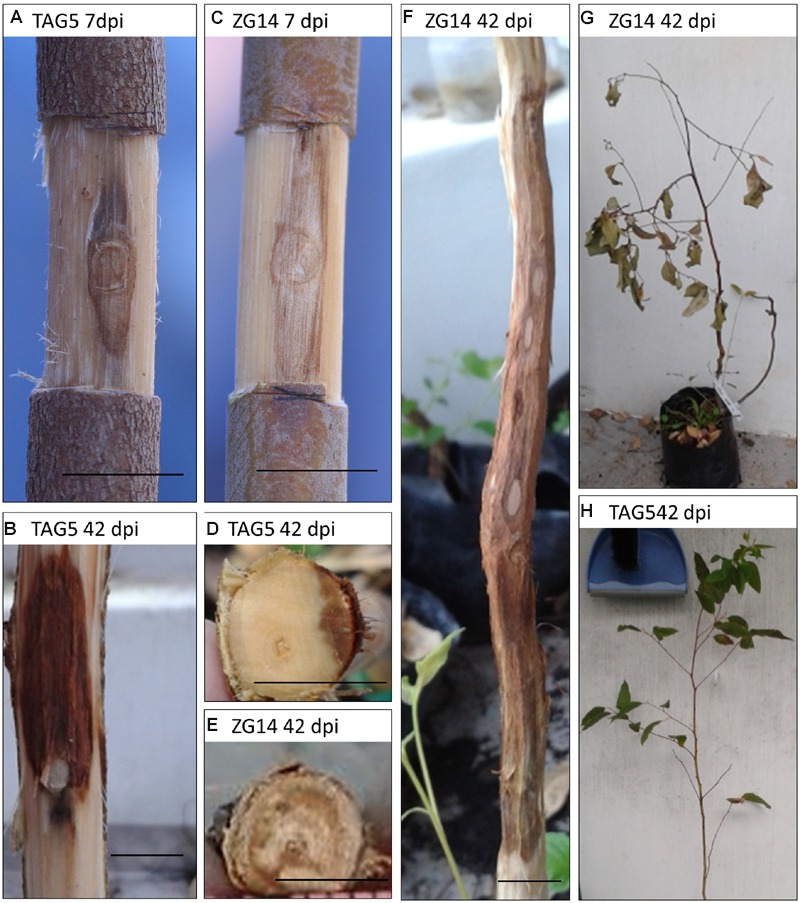
**Lesion development in *Eucalyptus grandis* clones inoculated with *C. austroafricana*.** Lesions in TAG5 **(A,B,D)** and ZG14 **(C,E,F)** at 7 dpi and 42 dpi are shown. Different rates of radial lesion development **(D,E)** were observed, and wilting occurred in some ZG14 plants by 42 dpi, while TAG5 leaves remained healthy **(G,H)**. Scale bars indicate 10 mm in all images except **(E)**, which indicates 5 mm.

The macroscopic changes in the stems of *E. grandis* clones during infection with *C. austroafricana* were documented over a period of 6 weeks. Inoculated ramets were compared to mock inoculated and unwounded plants. Characteristic brown lesions were observed from 3 dpi (days post-inoculation) to 42 dpi in both TAG5 (moderately resistant) and ZG14 (susceptible) plants (**Figure 2**) and were consistent with previous reports (Heath et al., 2006; Mangwanda et al., 2015). Lesion development occurred most rapidly in the axial direction, followed by radial and tangential spread (**Figure 2**). By 42 dpi, radial lesion development at the inoculation site was also slower in TAG5 than ZG14 (**Figures 2D,E**). In addition to lesion development, the inoculated plants exhibited an increasing number of symptoms indicative of wilting as infection progressed; the leaves of some ZG14 plants had died by 42 dpi, while the leaves of TAG5 plants were as healthy as controls (**Figures 2G,H**). No wilting was observed in wounded plants.

### The Location of *C. austroafricana* in Stem Tissue of Its Host, *E. grandis*

While the macroscopic symptoms of *C. austroafricana* stem canker disease in *Eucalyptus* have been observed previously, the physical interaction and spread of the pathogen within its host have not yet been described. In order to achieve this, sections of *E. grandis* stem tissue at the inoculation site were stained with lactophenol blue (LBO) or Safranin O and Fast Green, and examined using light microscopy.

**FIGURE 3:**
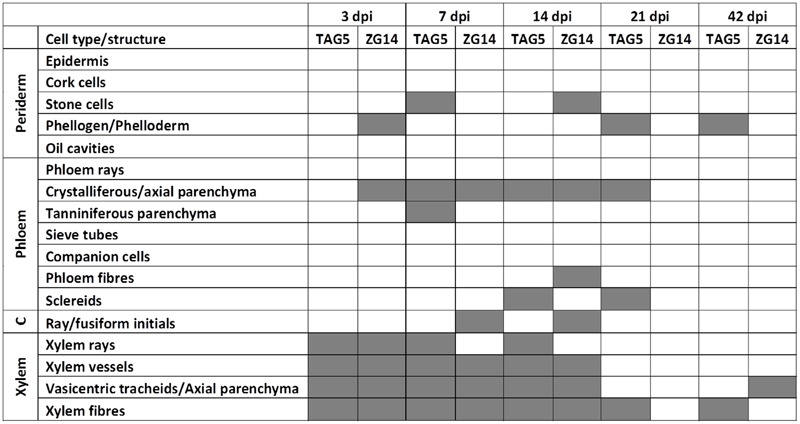
**A summary of the location of *C. austroafricana* within highly susceptible and moderately resistant *E. grandis* clones at the inoculation site using safranin/fast green or lactophenol blue staining.** C: cambium; dpi: days post-inoculation. Gray boxes indicate the presence of hyphae.

**FIGURE 4:**
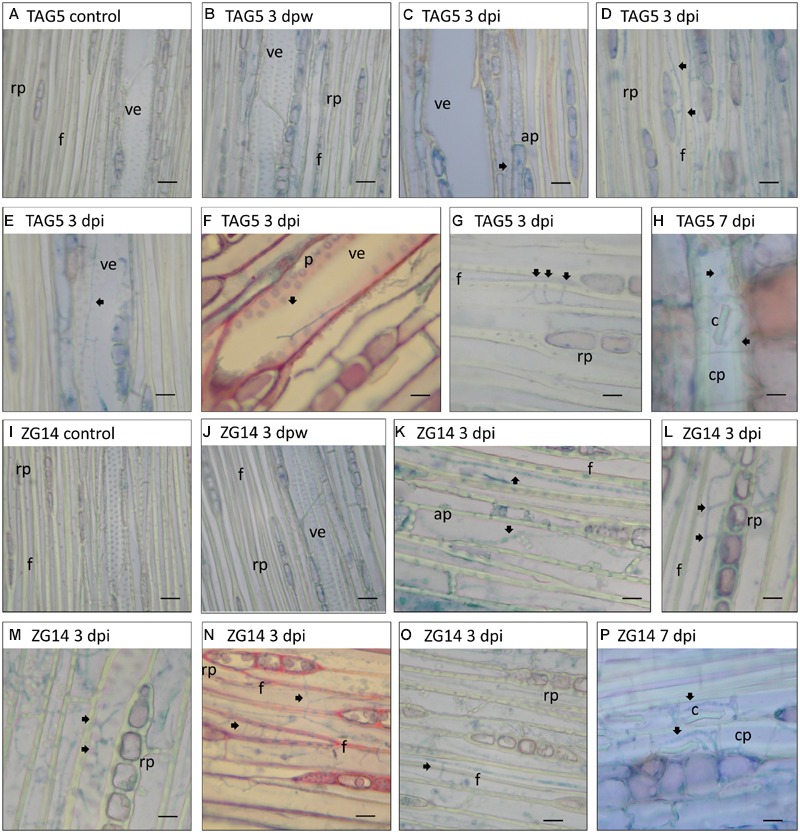
**Light micrographs of the most frequent sites where *C. austroafricana* was observed within inoculated TAG5 and ZG14 plants.** All sections were stained with lactophenol blue except **(F,N)**, which were stained with safranin/fast green. **(A–H)** TAG5. **(I–P)** ZG14. Arrows indicate hyphae. dpw: days post-wounding; dpi: days post-inoculation; rp: xylem ray parenchyma; f: xylem fiber; ve: xylem vessel element; ap: axial xylem parenchyma; p: cell wall pits; cp: phloem crystalliferous parenchyma; c: crystal. Scale bars: 10 μm.

The anatomy of the *E. grandis* plants was consistent with previous descriptions of this genus (Chattaway, 1953). Soon after inoculation (3 dpi), hyphae could be detected in xylem vessels, between xylem fibers, in axial xylem parenchyma/vasicentric tracheids, and in direct contact with xylem ray parenchyma cells and phloem crystalliferous parenchyma (**Figures 3** and **4**). In the bark, hyphae were most frequently seen in the phloem crystalliferous parenchyma (from 3 to 21 dpi in ZG14 and from 7 to 14 dpi in both clones, **Figures 4H,P**). However, the presence of hyphae in heavily pigmented or darkly stained phloem elements cannot be excluded. At 7 dpi, hyphae were also seen in tanniniferous phloem parenchyma, axial phloem parenchyma, and occasionally in peridermal stone cells. This distribution persisted until 14 dpi, at which point hyphae were also present in phloem fibers and sclereids. From 21 dpi, hyphae became less abundant in all of these cell types, and were rarely observed at the inoculation site by 42 dpi. By 21 dpi, most ray cells at the inoculation site appeared to have appeared to have died. Hyphae were occasionally observed in phellogen (3, 21, 42 dpi), phloem fibers (14 dpi in ZG14), and cambium (ZG14 7–14 dpi).

In TAG5 and ZG14, hyphae branched toward vascular ray parenchyma cells and occasionally other living cells. In several cases, a hypha would branch several times near ray parenchyma cells, appearing to enter each cell through the cell wall pits (**Figures 4D,L**). Hyphae also seemed to traverse adjacent vessel elements, fibers and cells via the pits (**Figures 4F,G,N,O**). The location of fungal hyphae coincided with the appearance of the lesion, suggesting that lesion development corresponds approximately to pathogen spread.

### Transcriptome Profiling of *C. austroafricana* Grown *In vitro* and *In planta*

To investigate the induction of pathogenicity factors in *C. austroafricana*, transcriptome profiling was performed for the fungus grown *in vitro* and *in planta* at 3 dpi (**Figure 1**). Transcriptome profiling of *C. austroafricana* grown *in vitro* yielded at least 32 million high-quality paired end reads per biological replicate. Of the 13,205 predicted gene models in the *C. austroafricana* genome, between 9,289 and 12,572 genes were expressed (FPKM > 0) under the different conditions (Supplementary Table S1). Quartile distributions of the range of FPKM values *in vitro* and *in planta* are indicated in Supplementary Table S2. Differential gene expression analysis with Cuffdiff identified 4312 genes that were significantly differentially expressed (DE) in minimal medium compared to complete medium (Supplementary Table S3). Within this list of DE genes, 2313 genes were up-regulated and 1999 genes were down-regulated in minimal medium. Expression analysis of the fungus grown *in planta* relative to the fungus grown on complete medium, identified 3689 (1774 up-regulated; 1915 down-regulated) and 3353 (1529 up-regulated; 1824 down-regulated) DE gene models in ZG14 and TAG5 respectively (Supplementary Tables S4 and S5). There were 1080 (389 up-regulated; 691 down-regulated) DE gene models expressed *in vitro* and *in planta*, while 1552 (718 up-regulated and 834 down-regulated) DE gene models were only expressed *in planta*. Additionally, 335 genes were only DE expressed in ZG14 (Supplementary Table S6).

### Gene-Ontology Enrichment of Differentially Expressed Genes

**FIGURE 5:**
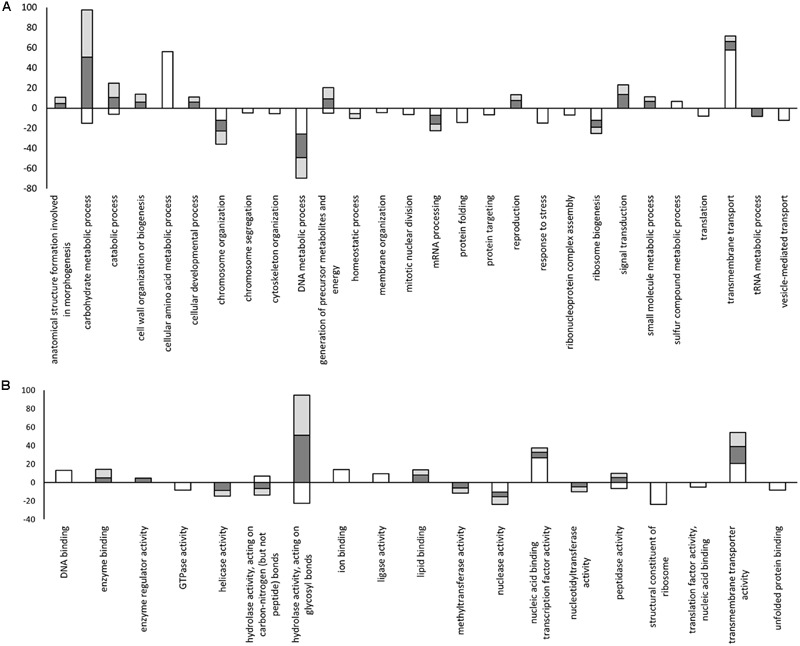
**Statistically enriched GO terms identified in the differentially expressed gene lists within the biological process and molecular function category. (A)** Biological process; **(B)** Molecular function. Positive values indicate over-represented terms and negative values indicate under-represented terms. White: minimal medium; Dark gray: ZG14; Light gray: TAG5. The y-axis represents the –log_2_(*p*-value) obtained with the Fisher’s exact test in Blast2GO. The x-axis = GO terms.

In order to identify biological processes that may contribute to pathogenicity, the enrichment of GO terms within each set of DE genes identified *in vitro* and *in planta* was investigated using Blast2GO. Terms from the biological process category that were unique to genes expressed *in planta* included “cell wall organization or biogenesis,” “cellular developmental process,” “reproduction,” “anatomical structure formation involved in morphogenesis,” and “signal transduction.” Biological process terms unique to genes expressed in minimal medium included “cellular amino acid metabolic process” and “sulfur compound metabolic process.” Interestingly, some terms within the biological process category such as “carbohydrate metabolic process,” “catabolic process,” and “generation of precursor metabolites and energy” were under-represented *in vitro* but over-represented *in planta* (**Figure 5A**).

In the molecular function category, the terms “hydrolase activity, acting on glycosyl bonds” and “peptidase activity” were over-represented *in planta* but under-represented *in vitro* (**Figure 5B**). Terms in the molecular function category that were unique to genes expressed under *in planta* conditions included “enzyme binding,” “lipid binding,” and “methyltransferase activity.” Molecular function terms unique to genes expressed *in vitro* included “ion binding” and “ligase activity.” For the cellular component category, more terms were found among the genes expressed *in vitro* compared to *in planta.* All the terms for the genes expressed *in vitro* were under-represented in the cellular component category. In this category, only one term, “extracellular region,” was over-represented and originated from the genes expressed *in planta* (**Supplementary Figure S1**).

### Identification of Genes Involved in Carbohydrate Metabolism

Due to the over-representation of the “carbohydrate metabolic process” term among the genes expressed *in planta*, the *C. austroafricana* genome was further examined for genes involved in carbohydrate metabolism. A total of 1525 candidates with homologous hits to known genes in the CAZyme database were identified. Among the fungal genes DE *in planta*, 465 candidates were expressed in TAG5, 525 in ZG14 and 554 *in vitro*. However, since all of the CAZyme database genes are not necessarily involved in pathogenicity, candidates specifically involved in cell wall hydrolysis and degradation were identified among the DE genes (**Table 1**). The list included xylanases, endoglucanases, polygalacturonases, cellulases, cellobiohydrolases, pectate lyases, and pectin methylesterases (PMEs). All of the genes involved in cell wall hydrolysis and/or degradation were either absent in fungus grown on MM or expressed at lower levels than *in planta*.

**Table 1:** Differentially expressed genes in *Chrysoporthe austroafricana* putatively involved in cell wall hydrolysis.

Gene ID^a^	Sequence description	CAZyme ID	CAZyme description	ZG14^b^	TAG5^b^	MM^b^
g11257	Cellobiohydrolase ii protein	GH6| CBM1	Cellobiohydrolase	6.66	5.48	–
g1627	Cellobiohydrolase ii	GH6| CBM1	Cellobiohydrolase	5.06	4.08	-3.36
g5329	Glycoside hydrolase family 5 protein	GH5	Cellulase	5.07	4.22	1.17
g4314	Endoglucanase 3 precursor	GH5	Cellulase	4.16	3.46	-0.98
g6568	Glycoside hydrolase family 5 protein	GH5	Cellulase	4.11	3.05	-2.64
g7427	Glycoside hydrolase family 5 protein	GH5	Cellulase	4.84	4.85	-1.37
g8109	Endo-*n*-acetyl-beta-d-glucosaminidase precursor	GH18	Chitinase	3.67	3.35	0.86
g4000	Class iii chitinase 2 protein	GH18| CBM1	Chitinase	2.76	3.17	2.90
g1953	Endochitinase 1 precursor	GH18	Chitinase	1.01	1.47	–
g1810	Endo- -beta-xylanase precursor	GH10| CBM1	Endo-1,4-β-xylanase	6.26	5.42	–
g5394	Glycoside hydrolase family 10 protein	GH10	Endo-1,4-β-xylanase	5.19	4.93	–
g5445	Xyloglucan-specific endo-beta- -glucanase a	GH12	Endoglucanase	5.86	6.07	–
g3498	Xyloglucan-specific endo-beta- -glucanase precursor	GH12	Endoglucanase	3.21	3.12	-1.17
g1022	Arabinogalactan endo- -beta-galactosidase	GH53	Endo-β-1,4-galactanase	5.00	5.35	0.92
g11198	Glycoside hydrolase family 7 protein	GH7	Endo-β-1,4-glucanase	7.46	6.82	0.91
g15	Cellobiohydrolase precursor	GH7	Endo-β-1,4-glucanase	7.12	7.05	–
g8111	Endoglucanase i	GH7	Endo-β-1,4-glucanase	3.27	1.94	-5.20
g1213	Achain pectin lyase a	PL1	Pectate lyase	2.34	2.51	–
g1125	Carbohydrate esterase family 8 protein	CE8	Pectin methylesterase	4.90	5.01	0.72
g11009	Pectinesterase precursor	CE8	Pectin methylesterase	3.29	3.46	-1.45
g9733	Glycoside hydrolase family 28 protein	GH28	Polygalacturonase	8.54	8.40	1.50
g3288	Endopolygalacturonase 1	GH28	Polygalacturonase	7.83	7.07	–
g2415	Glycoside hydrolase family 28 protein	GH28	Polygalacturonase	6.64	6.37	–
g12766	Rhamnogalacturonase a	GH28	Polygalacturonase	6.00	5.94	–
g1777	Exopolygalacturonase protein	GH28	Polygalacturonase	3.94	3.91	–
g4274	Pectin lyase-like protein	GH28	Polygalacturonase	2.97	3.68	–
g587	Galacturan -alpha-galacturonidase c	GH28	Polygalacturonase	2.05	2.23	–
g2053	Polygalacturonase 5	GH28	Polygalacturonase	1.30	1.45	-2.68
g3432	Endopolygalacturonase partial	GH28	Polygalacturonase	6.06	6.20	-1.59
g7776	Beta-glucosidase g	GH3	β-glucosidase	8.34	8.35	–
g1439	Glycoside hydrolase family 3 protein	GH3	β-glucosidase	6.02	6.04	–
g1799	Glycoside hydrolase family 3 protein	GH3	β-glucosidase	3.53	3.54	–
g5920	Glycoside hydrolase family 3 protein	GH3	β-glucosidase	1.97	2.64	–

*Expression levels are indicated as log_2_ values relative to complete medium. The minimal medium (MM) represents the *in vitro* expression data, whilst ZG14 and TAG5 represent the *in planta* expression data. ^a^Gene identities in the *C. austroafricana* genome. ^b^Expression values (log2) relative to complete medium obtained from Cuffdiff. Positive values indicate up-regulation; Negative values indicate down-regulation.*

### The Pathogen–Host Interaction (PHI) Database and Pathogenicity Genes

Since cell wall degradation is unlikely to be the only pathogenicity mechanism employed by *C. austroafricana*, we searched for additional candidates with known involvement in pathogenicity in the DE gene sets using a BLASTP analysis with the PHI database. A total of 1090 DE pathogenicity-related genes were identified among genes DE in ZG14, 971 genes in TAG5 and 1239 *in vitro*. The PHI database has a number of categories based on the phenotype of the mutants for genes involved in pathogenicity. We focused primarily on genes within the following categories: effectors, loss of pathogenicity and reduced virulence.

#### Effectors

This study identified 13 candidates that were homologous to known effectors in the PHI database (**Table 2**; Supplementary Table S7). Homologs included genes encoding an *Ace1, PemG1* and a protein containing a cerato-platanin domain from *Magnaporthe grisea*, as well as a *HopI1* from *Pseudomonas syringae*. Six *C. austroafricana* homologs to *ACE1* and one to a cerato-platanin *MgSM1* from *M. grisea*, as well as five homologs to *HopI1* from *P. syringae* were identified. Included in the list of genes homologous to the *Ace1* effectors were non-ribosomal peptide synthetases (nrps). One of these genes (g2672) with homology to an nrps-like enzyme was only expressed in ZG14 and TAG5 and not *in vitro*. A *HopI1* homolog, g3525, was expressed only in ZG14. Other effectors expressed specifically in ZG14 are shown in **Table 2**.

**Table 2:** Putative pathogenicity genes of *Chrysoporthe austroafricana* according to the Pathogen–Host Interaction (PHI) database.

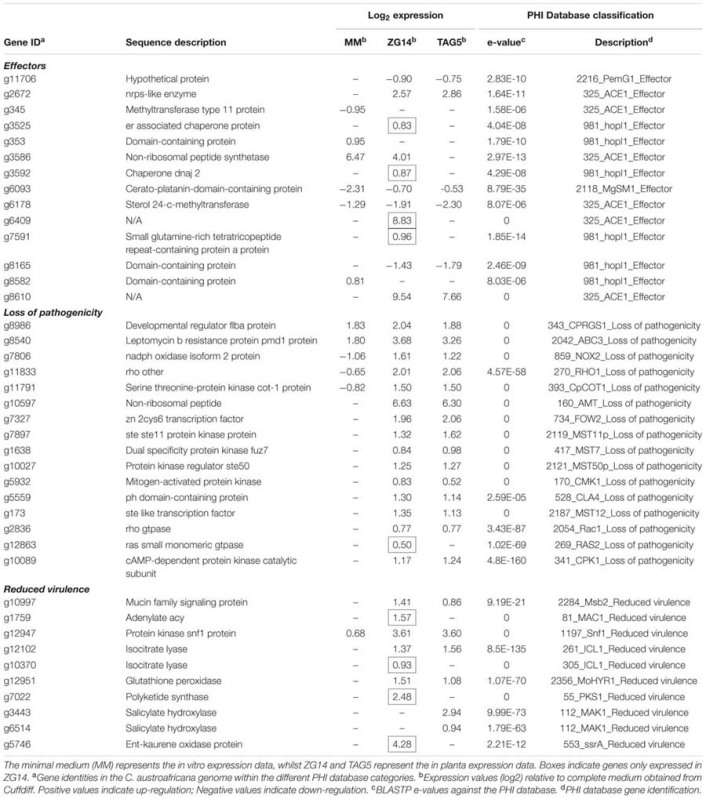

#### Loss of Pathogenicity

A total of 96 DE fungal genes in ZG14, 83 in TAG5 and 75 *in vitro* were homologous to genes in the “loss of pathogenicity” category in the PHI database. Mutants of these candidates in the PHI database had an apathogenic phenotype.

**FIGURE 6:**
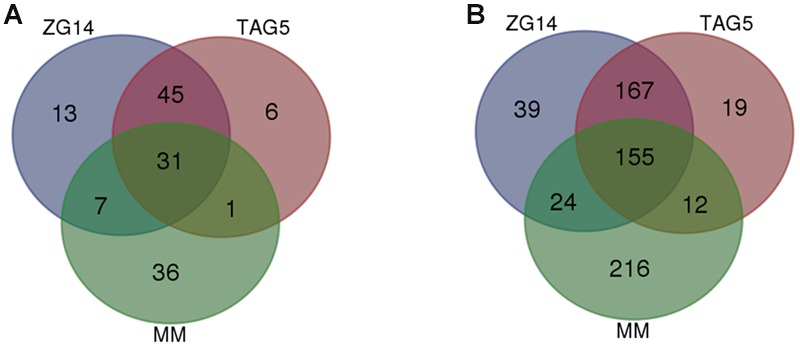
**Number of differentially expressed genes identified in the loss of pathogenicity and reduced virulence PHI categories in *E. grandis* clones TAG5 and ZG14. (A)** Loss of pathogenicity; **(B)** reduced virulence. MM, minimal medium.

A set of 31 DE genes was common to all conditions (**Figure 6A**; **Table 2**; Supplementary Table S7). One of the candidates that was expressed at approximately the same level in all conditions was g8986 (*developmental regulator flba protein*), a homolog of *Cryphonectria parasitica regulator of G-protein signaling* (CPRGS-1). Several homologs of membrane transporter genes from the ATP-binding cassette (ABC) and major facilitator superfamily (MFS) were DE *in vitro* and *in planta*, but with a larger fold change in the latter. These genes are associated with various cellular and metabolic functions and have been implicated in pathogenicity through the secretion of toxins. A candidate homologous to *ABC3* in the PHI database, g8540 (*leptomycin b resistance protein*), was up-regulated *in vitro* and *in planta*. In this study, g8540 was expressed at higher levels *in planta* (∼3-fold up-regulated) than *in vitro* (∼1.8 fold up-regulated). Among the genes that were down-regulated *in vitro* and up-regulated *in planta* were homologs of *NOX2* (g7806, *nadph oxidase isoform 2 protein*), *RHO1* (g11833, *rho*) and *COT1* (g11791, *serine threonine-protein kinase cot-1 protein*).

Forty-five candidates with homology to the “loss of pathogenicity” category in the PHI database were only expressed *in planta* (**Figure 6A**). Among these candidates were an *nrps* gene (g10597, *non-ribosomal peptide*) homologous to *AMT1* in the PHI database, and a homolog of transcription regulator *FOW2* [g7327, *Zn(II) 2cys6 transcription factor*], which is involved in mediating plant infection in *Fusarium oxysporum*. This set included multiple genes involved in the mitogen activated protein kinase (MAPK) and cAMP signaling cascades. The MAPK cascade is involved in diverse cellular functions. Our candidates included the backbone of this signaling cascade *Ste11* (MAPKKK), the adaptor protein *Ste50, Ste7* (MAPKK), *Fus3/Kss1* (MAPK), upstream candidates (*Ste20* and *Cla4*) and downstream candidates (*Ste12*). *Ste12* controls MAPK responses pertaining specifically to invasive growth and pathogenicity. Induction of the MAPK cascade affecting pathogenicity is initiated by a small GTPase, *Rac1.* All of these MAPK signaling cascade candidates were up-regulated only *in planta* (**Table 2**; Supplementary Table S7). The expression of *RAS2* (g12863, *ras small monomeric gtpase*), which acts upstream of *Ste20*, was DE only in ZG14 (**Table 2**; Supplementary Table S7). Furthermore, the cAMP cascade involves three main components: heteromeric G-proteins, adenylate cyclase (AC) and cAMP-dependent protein kinase (CPK). *CPK* (g10089, *camp-dependent protein kinase catalytic subunit*) was DE in both TAG5 and ZG14.

#### Reduced Virulence

In addition to the “loss of pathogenicity” category, 385 genes homologous to candidates associated with a reduced virulence phenotype were identified in ZG14, 355 in TAG5 and 407 *in vitro* (**Figure 6B**; **Table 2**; Supplementary Table S7). This category includes genes that facilitate enhanced virulence, i.e., mutants have a reduction in virulence, but do not completely lose their pathogenicity. A transmembrane domain containing gene encoding a homolog of mucin (g10997, *Msb2*), which is involved in initiating the MAPK cascade via *Rac1*, was found in this category. Expression of a homolog of *Msb2* was up-regulated only *in planta*, as observed with the other MAPK cascade genes. A homolog of the AC (g1759, *adenylate cyclase, acy*) component of the cAMP signaling pathway was also in this category and only up-regulated in ZG14. Several genes involved in cell wall hydrolysis were identified in these lists, including homologs of *pectinesterase, endo-beta xylanase, polygalacturonase and glucan beta-glucosidase*. A *Snf1* homolog (g12947, *protein kinase snf1 protein*) was expressed at lower levels *in vitro* (∼0.6 fold up-regulated) than *in planta* (∼3.6 fold up-regulated) and is associated with the regulation of CWDEs.

Other candidates with homologs in this category included *isocitrate lyase* (*ICL1*), which is involved in the glyoxylate cycle, a *glutathione peroxidase* (*MoHYR1*) involved in ROS detoxification, *polyketide synthases* (*PKSs*) involved in toxin production, and numerous MFS and ABC transporters (**Table 2**; Supplementary Table S7). In *C. austroafricana, ICL* (g12102, *isocitrate lyase*) was DE only *in planta*. An additional *ICL* homolog (g10370, *isocitrate lyase*) was DE only in ZG14. The *HYR1* gene (g12951) was DE only *in planta*, with a larger fold change in the susceptible host. *PKS* genes of *C. austroafricana* (g11676 and g7022) were DE only in ZG14, but only g7022 was associated with reduced virulence.

Genes associated with the manipulation of phytohormone signaling, particularly salicylic acid and gibberellins, were also present in the “reduced virulence” category (**Table 2**; Supplementary Table S7). In *C. austroafricana*, two *salicylate hydroxylase* genes (g3443 and g6514) associated with reduced virulence were expressed only in TAG5. An additional putative *salicylate hydroxylase* gene (g11021) was up-regulated with a larger fold change *in planta* (∼3 fold up-regulated) than *in vitro* (∼0.7 fold up-regulated), but this gene was associated with an “unaffected pathogenicity” phenotype. A *C. austroafricana ent-kaurene oxidase* gene (g6592, *ent-kaurene oxidase*) was DE in TAG5 and ZG14, while another (g5746, ent-kaurene oxidase protein) was only expressed in ZG14. The homolog of the gene expressed only in ZG14 was associated with a reduced virulence phenotype in the PHI database.

### RT-qPCR Validation of Transcriptome Data

**FIGURE 7:**
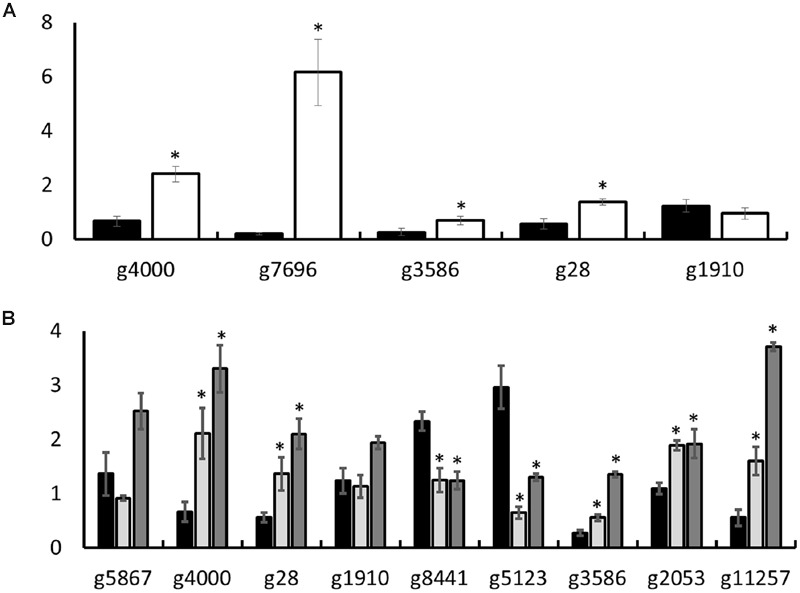
**Reverse transcription quantitative polymerase chain reaction (RT-qPCR) validation of transcriptome profiling *in vitro* and *in planta*. (A)** *In vitro*; **(B)** *in planta*. The y-axis indicates the expression level. Black bars represent complete medium. **(A)** The white bars represent minimal medium. **(B)** The light gray bars represent TAG5 and the dark gray bars represent ZG14. Significance relative to complete medium is indicated by (Student’s *t*-test, two-tailed, ^∗^*p*-value < 0.05).

Selected candidates from among the genes DE in minimal medium, TAG5, and ZG14 were used to validate the transcriptome profiling with reverse transcription quantitative polymerase chain reaction (RT-qPCR). The following genes were chosen for validation of expression *in vitro* (complete medium vs. minimal medium) well as *in planta* (complete medium vs. ZG14; complete medium vs. TAG5): g4000 (*class iii chitinase 2 protein*), g3586 (*non-ribosomal peptide synthetase*), g28 (*polyamine transporter 2 protein*), and g1910 (*mfs transporter*). In addition, g7696 (*monocarboxylate permease-like protein*) was selected for validation of genes expressed *in vitro*. The following genes were also selected for the validation of genes expressed *in planta*: g5867 (*acetolactate synthase protein*); g8441 (*auxin-induced protein*); g2053 (*polygalacturonase 5*) and g11257 (*cellobiohydrolase ii protein*). All the genes, except g1910, matched their corresponding transcriptome profiles (**Figure 7**).

## Discussion

The pathogen *C. austroafricana* naturally infects *E. grandis* through wounds in the stem, but this infection mechanism can be mimicked under controlled conditions in order to study the interaction. In our study, this established pathosystem was employed to understand how the pathogen spreads through the host and to identify which pathogenicity factors are activated by *C. austroafricana* in order to propose mechanisms by which it can cause disease.

### The Physical Interaction between *C. austroafricana* and *E. grandis*

Tissue of moderately resistant and highly susceptible *E. grandis* clones infected with *C. austroafricana* was subjected to histochemical analysis in order to visualize the physical interaction of this pathogen with its host. The pathogen was observed in the same cell types and structures in both clones. This suggests that it follows the same route of spread in these clones. This route may simply be that which presents the least resistance to movement. For example, wood-rotting fungi typically spread faster axially, since most large elements are orientated in this direction (Pearce, 1996). In this study, hyphae were most frequently seen in non-living elements such fibers and xylem vessels. These elements would enable axial spread; fiber lumina are long, while xylem vessels form continuous channels of relatively large diameter through which hyphae can spread with ease. Radial spread could be facilitated by ray parenchyma cells, where hyphae were also observed, and through the walls of vessel elements. Tangential spread would involve movement through the cell wall pits of adjacent elements such as xylem vessels and fibers, and is expected to be most arduous (Pearce, 1996). These rates of spread are consistent with the rate of lesion development and with the tentative observation that lesion formation coincides with the location of hyphae.

In addition, hyphae showed a marked affinity for cell wall pits. Hyphae were observed in direct contact with cell wall pits, which they frequently traversed, and also appeared to enter living parenchyma cells via cell wall pits. The fungus could obtain nutrients from these host cells or manipulate the plant defense response during this direct interaction. The ability to cross cell wall pits may be crucial for *C. austroafricana* to proliferate in the host. Knowledge about pathogen location and putative mechanism of spread provides a foundation for exploring the molecular mechanisms by which the pathogen proliferates in the host.

### Minimal Medium Reveals General Stress Responses Induced in *C. austroafricana*

The stress induced by the limited availability of nutrients in MM resulted in responses that could be related to pathogenicity mechanisms. Signal transduction is crucial for the response of eukaryotic cells to extracellular signals. Candidates such as the G-protein *CRPGS-1* are expected to be DE *in vitro* and *in planta*, and this was observed. In *Cryphonectria parasitica, CPRGS-1* has a crucial role in pathogenicity, since loss of function mutants are unable to infect chestnut stems (Lengeler et al., 2000; Segers et al., 2004). In this study, homologs of several genes that allow the pathogen to survive oxidative stress and facilitate the maintenance of fungal cell wall integrity were either down-regulated *in vitro* or more highly induced *in planta* (**Table 2**). Numerous studies showed that mutations in these genes result in apathogenicity (Scheffer et al., 2005; Sun et al., 2006; Egan et al., 2007; Martinez-Rocha et al., 2008; Segmüller et al., 2008; Tudzynski et al., 2012; Morita et al., 2013). These results show that the responses in minimal medium can provide an indication of pathogenicity mechanisms, but cannot replace an investigation of the direct interaction *in planta*.

### Pathogenicity Mechanisms of *C. austroafricana* Elucidated *In planta*

#### Cell Wall Degradation and Fungal Cell Wall Maintenance

Investigating the expression of fungal genes *in planta* enables identification of putative fungal pathogenicity mechanisms induced in the host environment. One of the first events during fungal invasion is the secretion of CWDEs to permeate the host tissue (Kubicek et al., 2014). We identified several putative CWDEs that were up-regulated *in planta* (**Table 1**). A key candidate involved in the regulation of CWDEs, *Snf1*, is an important pathogenicity factor in *Magnaporthe oryzae, Fusarium oxysporum, Gibberella zeae*, and *Cochliobolus carbonum* (Tonukari et al., 2000; Ospina-Giraldo et al., 2003; Lee et al., 2009; Yi et al., 2009; Turra et al., 2014). *Snf1* was expressed at higher levels *in planta* than *in vitro* in the current study (**Table 2**). These CWDEs could be crucial for the movement of *C. austroafricana* via cell wall pits, allowing the pathogen to manipulate or penetrate host cells.

#### Signal Transduction

In several eukaryotic organisms, signal transduction occurs via the sequential phosphorylation of protein kinases known as MAPKs. Fungal pathogens predominantly employ the Fus3/Kss1 pathway for mediating pathogenesis, and orthologs of candidates in this pathway have been found in several pathogenic fungi (Zhao et al., 2007). In this study, the ortholog of Fus3/Kss1 (g5932, mitogen-activated protein kinase) was identified as CMK1 (Takano et al., 2000). RAS2 was DE in ZG14 (**Table 2**). Deletion of *RAS2* in *F. graminearum* decreased phosphorylation in the Fus3/Kss1 pathway, compromising virulence (Bluhm et al., 2007; Turra et al., 2014). *AC* is also expressed only in ZG14. Candidates of the cAMP signaling cascade, *CPK* and *AC*, have been associated with pathogenicity in various phytopathogen interactions (Choi and Dean, 1997; Klimpel et al., 2002; Yamauchi et al., 2004; Wayne and Rollins, 2007; Garcia-Martinez et al., 2012; Bormann et al., 2014).

#### Fungal Effectors and Toxin Production

Upon infiltration, the fungus needs to contend with the defense mechanisms induced by the host. For successful colonization, the fungus requires various pathogenicity mechanisms such as the secretion of effector molecules (Stergiopoulos and de Wit, 2009; Wang et al., 2014). Some effector proteins, the NRPSs, exert their role in pathogenicity by producing fungal toxins (Oide et al., 2006; Chen et al., 2013; Bills et al., 2014). In this study, we identified two NRPS candidates (**Table 2**). PKSs are also associated with the biosynthesis of toxins in *Cochliobolus heterostrophus, Cercospora nicotianae, Botrytis Cinerea*, and *Gibberella fujikuroi* (Yang et al., 1998; Proctor et al., 1999; Choquer et al., 2005; Dalmais et al., 2011). In this study, the expression of two PKS genes only in the susceptible host environment may contribute to the proliferation of *C. austroafricana*.

#### Combating Host Responses and Nutrient Assimilation

Phytohormone signaling cascades are important for several defense mechanisms that can lead to slower spread of the pathogen. These pathways can be manipulated by pathogens to facilitate their own growth and proliferation. Fungi can produce hormones to perturb the phytohormone balance within the host to facilitate susceptibility (Mobius and Hertweck, 2009). One of the first committed intermediates for GA biosynthesis is ent-kaurene, which is oxidized to ent-kaurenol by ent-kaurene oxidase (Tudzynski, 2005). Ent-kaurene is a common intermediate in GA biosynthesis of plants and fungi (Hedden et al., 2002). In *Xanthomonas oryzae*, ent-kaurene oxidase acts as a virulence factor by manipulating the interplay between GA and JA in rice (Lu et al., 2015). In TAG5, GA levels were reduced at 3 dpi whilst this reduction was only observed in the susceptible host at 7 dpi (Mangwanda et al., 2015). This may be due to the expression of an *ent-kaurene oxidase* candidate in the susceptible ZG14 at 3dpi (**Table 2**). Furthermore, salicylate hydroxylase is an SA-degrading enzyme that converts SA to catechol and can serve as a virulence factor in pathogens that require lower levels of SA to proliferate within maize plants (Penn and Daniel, 2013; Rabe et al., 2013). We previously showed that TAG5 has higher basal levels of SA than ZG14 (Mangwanda et al., 2015) and that the pre-treatment of ZG14 with SA increases its level of resistance to that of TAG5 (Naidoo et al., 2013). Therefore, the expression of putative salicylate hydroxylases in TAG5 (**Table 2**) is another possible mechanism by which the pathogen manipulates the host defense response. Such potential interactions, i.e., ent-kaurene oxidase modulating GA and SA hydroxylase modulating SA, have been described in herbaceous systems. Our findings support the notion that these parallel mechanisms exist in the interaction of pathogens with herbaceous and woody plants.

Phytopathogens invade host cells to obtain nutrients which aid in establishing infection. One of the strategies that fungi employ to achieve this is through the glyoxylate pathway, which contains the key enzyme ICL. This enzyme contributes to pathogenicity in pathogens such as *Leptosphaeria maculans, Colletotrichum lagenarium*, and *M. grisea* (Idnurm and Howlett, 2002; Wang et al., 2003; Asakura et al., 2006; Dunn et al., 2009). In the current study, we observed the expression of ICL *in planta* and not *in vitro*, suggesting a role for the glyoxylate pathway in *C. austroafricana* virulence.

## Conclusion

We have identified possible mechanisms and candidate genes associated with fungal spread and pathogenicity of *C. austroafricana* in *E. grandis*. While the PHI database contains data from functionally characterized plant pathogen interactions, pending functional characterization, the roles of the genes in the *C. austroafricana* interactions are speculative. Future work will focus on the putative *salicylate hydroxylase* and *ent-kaurene oxidase* genes in *C. austroafricana* to determine their roles in pathogenicity. This will provide functional evidence of whether these host defense and pathogen strategies are similar in herbaceous and woody pathosystems.

## Author Contributions

RM performed the transcriptome experimental work and associated data analysis and interpretation, as well as writing the manuscript. LZ conceived and performed the microscopic investigation and contributed to writing the manuscript. SN conceived the study, obtained funding to support the research, participated in its design, coordination, biological interpretation of the results and helped to write the manuscript. AM, NvdM, LM, and DB provided input into the design and technical aspects, and assisted with critical evaluation of the manuscript. All authors have read and approved the final version of the manuscript.

## Conflict of Interest Statement

The authors declare that the research was conducted in the absence of any commercial or financial relationships that could be construed as a potential conflict of interest.

## Footnotes

1 http://www.cazy.org

2 www.clcbio.com

## Supplementary Material

FIGURE S1 **Statistically enriched GO terms identified in the differentially expressed gene lists within the cellular component category.** Positive values indicate over-represented terms and negative values indicate under-represented terms. White: minimal medium; Dark gray: ZG14; Light gray: TAG5. The y-axis represents the –log_2_(*p*-value) obtained with the Fisher’s exact test in Blast2GO. The x-axis represents the GO terms.
