# Supplementary figures and images for: Localization and Transcriptional Responses of Chrysoporthe austroafricana in Eucalyptus grandis Identify Putative Pathogenicity Factors
Source: Front Microbiol. 2016 Dec 8;7:1953. doi: 10.3389/fmicb.2016.01953 (PMC5143476; doi:10.3389/fmicb.2016.01953)

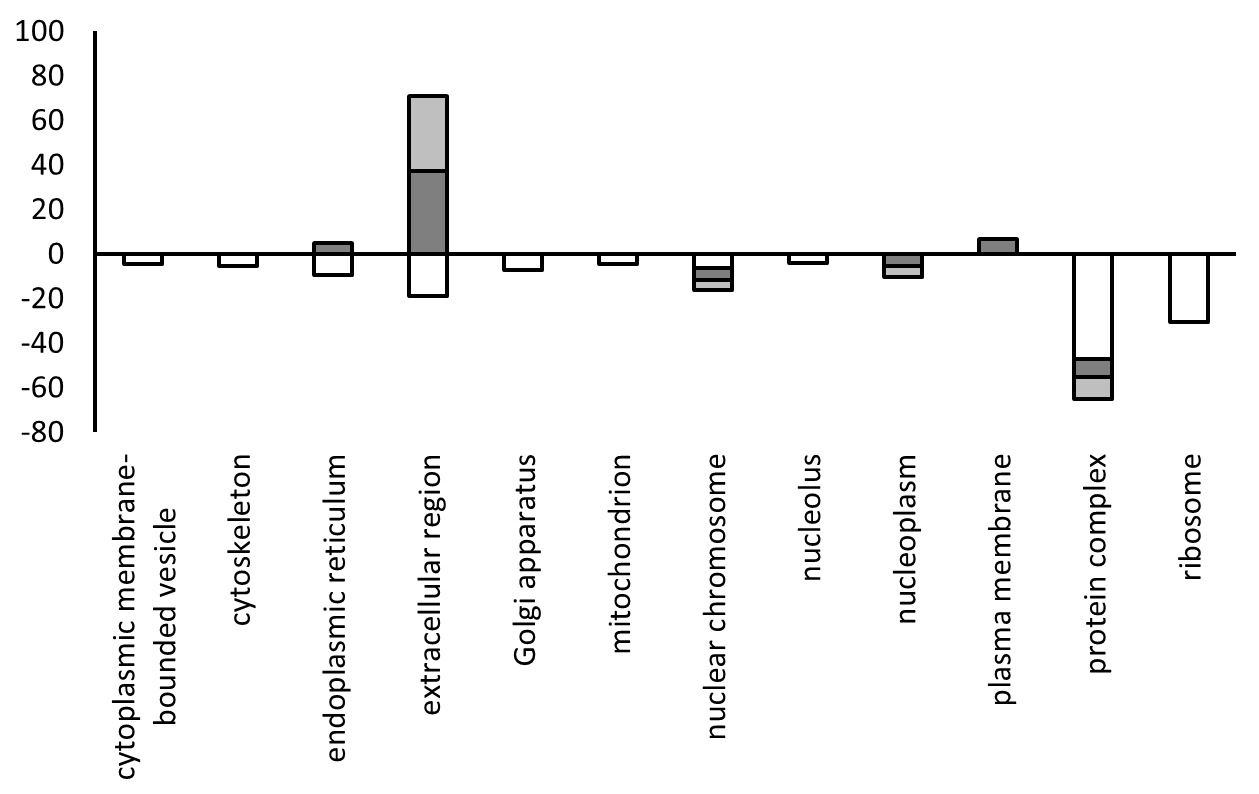

Supplement: FIGURE S1 — Statistically enriched GO terms identified in the differentially expressed gene lists within the cellular component category. Positive values indicate over-represented terms and negative values indicate under-represented terms. White: minimal medium; Dark gray: ZG14; Light gray: TAG5. The y-axis represents the –log2(p-value) obtained with the Fisher’s exact test in Blast2GO. The x-axis represents the GO terms. [file Image_1.TIF]
