# Supplementary material for: Localization and Transcriptional Responses of Chrysoporthe austroafricana in Eucalyptus grandis Identify Putative Pathogenicity Factors
Source: Front Microbiol. 2016 Dec 8;7:1953. doi: 10.3389/fmicb.2016.01953 (PMC5143476; doi:10.3389/fmicb.2016.01953)
Supplement: Supplementary file 2 [file Table_1.DOCX]

**Table S1**: Summary statistics for transcriptome profiling of *Chrysoporthe austroafricana*

| **Samples** | **RIN** | **Total PE Reads** | **% Mapped reads to *C. austroafricana*** | | | **% Mapped reads to *E.grandis genome*** | **Q20 Percentage (%)** | **GC Percentage (%)** | **Expressed genes** |
| --- | --- | --- | --- | --- | --- | --- | --- | --- | --- |
|  |  |  | Paired | Singletons | Total |  |  |  |  |
| 1. Complete Media_BR1 | 7.9 | 32.141161 | 94.0 | 1.9 | 95.9 | - | 98.1 | 56.84 | 12477 |
| 2. Complete Media_BR2 | 7.6 | 30.008628 | 94.5 | 1.9 | 96.4 | - | 98.14 | 56.38 | 12434 |
| 3. Complete Media_BR3 | 7.8 | 34.342786 | 94.3 | 1.8 | 96.1 | - | 98.07 | 57.19 | 12477 |
| 4. Minimal media_BR1 | 9.1 | 33.951504 | 93.3 | 2.0 | 95.4 | - | 98.03 | 57.19 | 12498 |
| 5. Minimal media_BR2 | 8.7 | 34.477061 | 93.8 | 1.9 | 95.7 | - | 98.06 | 57.16 | 12572 |
| 6. Minimal media_BR3 | 6.9 | 34.256842 | 94.3 | 1.9 | 96.2 | - | 98.04 | 57.07 | 12520 |
| 7. TAG5 Infected_BR1 | 9.6 | 37.617103 | 1.6 | 0.5 | 2.1 | 75.5 | 97.75 | 48.67 | 9166 |
| 8. TAG5 Infected_BR2 | 9.5 | 37.390551 | 2.7 | 0.5 | 3.2 | 76.9 | 97.58 | 49.31 | 9609 |
| 9. TAG5 Infected_BR3 | 9.5 | 38.684116 | 1.5 | 0.4 | 1.9 | 78.6 | 97.66 | 49.41 | 9067 |
| 10. ZG14 Infected_BR1 | 9.7 | 38.062937 | 2.4 | 0.5 | 2.9 | 75 | 98.13 | 49.33 | 9412 |
| 11. ZG14 Infected_BR2 | 9.6 | 34.373634 | 2.7 | 0.6 | 3.2 | 76.2 | 98.18 | 48.36 | 9289 |
| 12. ZG14 Infected_BR3 | 9.7 | 36.626103 | 2.7 | 0.5 | 3.2 | 75.8 | 98.13 | 48.77 | 9500 |
