# Supplementary material for: Localization and Transcriptional Responses of Chrysoporthe austroafricana in Eucalyptus grandis Identify Putative Pathogenicity Factors
Source: Front Microbiol. 2016 Dec 8;7:1953. doi: 10.3389/fmicb.2016.01953 (PMC5143476; doi:10.3389/fmicb.2016.01953)
Supplement: Supplementary file 3 [file Table_2.DOCX]

**Table S2: Quartile distribution of FPKM values for *Chrysoporthe austroafricana* across the datasets *in vitro* and *in planta***

|  | ***In vitro*** | | | | | | ***In planta*** | | | | | |
| --- | --- | --- | --- | --- | --- | --- | --- | --- | --- | --- | --- | --- |
|  | **CM1** | **CM2** | **CM3** | **MM1** | **MM2** | **MM3** | **ZG14_BR1** | **ZG14_BR2** | **ZG14_BR33** | **TAG5_BR1** | **TAG5_BR2** | **TAG5_BR3** |
| **Minimum** | 0 | 0 | 0 | 0 | 0 | 0 | 0 | 0 | 0 | 0 | 0 | 0 |
| **25%** | 1.89 | 1.77 | 1.89 | 2.13 | 2.47 | 1.95 | 0 | 0 | 0 | 0 | 0 | 0 |
| **Median (50%)** | 9.06 | 8.83 | 9.86 | 11.44 | 12.19 | 9.95 | 7.33 | 7.36 | 7.80 | 7.49 | 8.21 | 7.63 |
| **75%** | 27.49 | 25.70 | 28.97 | 30.75 | 32.64 | 30.22 | 24.59 | 24.10 | 24.29 | 24.13 | 25.49 | 23.76 |
| **Maximum** | 56467.90 | 52639.70 | 47095.90 | 51668.00 | 45705.60 | 45733.60 | 32833.60 | 48860.00 | 25688.20 | 291574.00 | 67174.40 | 95572.50 |
