# Supplementary material for: Localization and Transcriptional Responses of Chrysoporthe austroafricana in Eucalyptus grandis Identify Putative Pathogenicity Factors
Source: Front Microbiol. 2016 Dec 8;7:1953. doi: 10.3389/fmicb.2016.01953 (PMC5143476; doi:10.3389/fmicb.2016.01953)
Supplement: Supplementary file 7 [file Table_6.DOCX]

**Table S6**: **Degree of overlap within the differentially expressed genes *in vitro* and *in planta*.** Minimal media (MM) represents *in vitro*.

| **Category** | | |  |
| --- | --- | --- | --- |
| **MM** | **TAG5** | **ZG14** | **Number of genes** |
|  |  |  | 849 |
|  |  |  | 37 |
|  |  |  | 691 |
|  |  |  | 1 |
|  |  |  | 22 |
|  |  |  | 1 |
|  |  |  | 266 |
|  |  |  | 69 |
|  |  |  | 63 |
|  |  |  | 1579 |
|  |  |  | 11 |
|  |  |  | 150 |
|  |  |  | 40 |
|  |  |  | 2 |
|  |  |  | 389 |
|  |  |  | 37 |
|  |  |  | 105 |
|  |  |  | 98 |
|  |  |  | 834 |
|  |  |  | 1 |
|  |  |  | 88 |
|  |  |  | 2 |
|  |  |  | 718 |
|  |  |  | 126 |
|  |  |  | 229 |
