# Supplementary material for: Localization and Transcriptional Responses of Chrysoporthe austroafricana in Eucalyptus grandis Identify Putative Pathogenicity Factors
Source: Front Microbiol. 2016 Dec 8;7:1953. doi: 10.3389/fmicb.2016.01953 (PMC5143476; doi:10.3389/fmicb.2016.01953)
Supplement: Supplementary file 8 [file Table_7.DOCX]

**Table S7. Dehydration and wax infiltration of stem samples*.*** Dehydration solutions were diluted in distilled water.

| **Dehydration solution** | **Incubation** |
| --- | --- |
| 25% butanol, 30% ethanol | 60 minutes |
| 40% butanol, 30% ethanol | 60 minutes |
| 55% butanol, 25% ethanol | 60 minutes |
| 70% butanol, 20% ethanol | 120 minutes |
| 85% butanol, 15% ethanol | 120 minutes |
| 100% butanol | 60 minutes |
| **Wax infiltration** |  |
| 50% wax, 50% butanol | Overnight, 60 °C |
| 100% wax | Until butanol evaporates, 60 °C |
